# Mid Holocene rapid thinning and rethickening of the East Antarctic ice sheet suggested by glacial isostatic adjustment

**DOI:** 10.1038/s41598-025-24176-4

**Published:** 2025-11-17

**Authors:** Jun’ichi Okuno, Akihisa Hattori, Koichiro Doi, Yuichi Aoyama, Yoichi Fukuda

**Affiliations:** 1 Joint Support-Center for Data Science Research, Research Organization of Information and Systems, Midori-cho 10-3, Tachikawa-shi, 190-8518 Japan; 2 National Institute of Polar Research, Midori-cho 10-3, Tachikawa-shi, 190-8518 Japan; 3 The Graduate University for Advanced Studies, SOKENDAI, Midori-cho 10-3, Tachikawa-shi, 190-8518 Japan; 4 Kyoto University, Yoshida-honmachi, Sakyo-ku, Kyoto-shi, 606-8501 Japan

**Keywords:** Palaeoclimate, Geophysics

## Abstract

Understanding East Antarctic Ice Sheet dynamics during the mid-Holocene remains challenging due to complex climate interactions. Using Global Navigation Satellite System observations and glacial isostatic adjustment modeling, we investigate the ice-sheet history in the Lützow–Holmbukta region. Here we show that scenarios incorporating modest (65–100 m) ice-sheet re-thickening following previously documented rapid thinning of ~ 400 m between 9 and 6 ka provide improved agreement with geodetic observations compared with global deglaciation models. Our analysis constrains the rheological structure beneath East Antarctica, suggesting a lithospheric thickness of 50–70 km, upper mantle viscosities of 5–7 × 10^20^ Pa s, and lower mantle viscosities of 6–80 × 10^21^ Pa s, which show excellent agreement with independent constraints from effective elastic thickness studies and are broadly compatible with seismological estimates. These findings highlight the need to incorporate regional ice-sheet histories when investigating past ice-sheet dynamics, which is essential for understanding present-day behavior and predicting future responses to climate change.

**Supplementary Information:**

The online version contains supplementary material available at 10.1038/s41598-025-24176-4.

## Introduction

Climatological constraints on the mid-Holocene period (8–5 ka) are crucial for understanding Earth’s climate system and its response to natural forcing. Recent studies have provided insights into fluctuations in ice-sheet thickness and extent during this time, particularly in East Antarctica. While substantial evidence exists for widespread ice-sheet retreat during this period, the potential for subsequent stabilization or modest re-thickening remains an area of active investigation. Kawamata et al. (2020)^1^ investigated rocks in the Skarvsnes area along the Lützow–Holmbukta coast and found evidence of ~ 400 m of rapid ice-sheet thinning between 9 and 6 ka. This raises the question as to whether ice-sheet retreat of a similar scale occurred in other parts of East Antarctica during the mid-Holocene. Suganuma et al. (2022)^2^ addressed this question by studying the glacial history of central Dronning Maud Land, located to the west of the Lützow–Holmbukta coast. Their results revealed rapid thinning of the ice sheet and widespread deglaciation during the mid-Holocene, indicating that ice-sheet retreat along the Lützow–Holmbukta coast was part of a larger-scale event. Furthermore, Jones et al. (2022)^3^ reviewed the geological evidence for Holocene fluctuations in the Antarctic Ice Sheet (AIS) and proposed that mid-Holocene ice-sheet retreat may have occurred across the entire continent, thereby highlighting the need for further research to understand the drivers and implications of these changes.

The dynamics of the AIS during the Holocene is a subject of intense debate and provides insights into ice-sheet behavior in response to climate change. Recent studies have revealed a complex pattern of changes in ice-sheet elevation, characterized by substantial lowering followed by localized readvance. This pattern challenges earlier, simpler models of post-glacial ice-sheet retreat and highlights the dynamic nature of ice-sheet response to climatic forcing. Extensive research, including geomorphological, ice core, and numerical modeling studies, has shown the AIS experienced a substantial lowering of ice surface elevation following the Last Glacial Maximum. Bentley et al. (2014)^4^ and Stone et al. (2003)^5^ reported a substantial ice-sheet retreat in West Antarctica at ca. 9 ka, with ice surface lowering of >100 m in some areas. Similarly, Mackintosh et al. (2011)^6^ and Jones et al. (2015)^7^ documented retreat and thinning in East Antarctica during the early Holocene. However, the timing and magnitude of these changes are spatially variable. Whitehouse et al. (2012)^8^ and Mackintosh et al. (2014)^9^ identified regional differences in the onset of major ice-sheet retreat, ranging from approximately 15 to 8 ka in different sectors of Antarctica. This variability highlights the effects of local factors, such as glacier bed topography, ocean circulation, and atmospheric conditions, on ice-sheet dynamics. Intriguingly, recent studies have provided evidence of ice-sheet readvance following a period of retreat. Siegert et al. (2013)^10^ and Bradley et al. (2015)^11^ suggested that parts of the West Antarctic Ice Sheet began to readvance at ca. 4 ka. Most notably, Kingslake et al. (2018)^12^ presented evidence of extensive retreat and subsequent readvance of the West Antarctic Ice Sheet during the middle to late Holocene, indicating the ice sheet retreated beyond its current position before readvancing over the past few thousand years. Recent studies have further enhanced this understanding, with Johnson et al. (2022)^13^ providing a comprehensive review of the evidence for Holocene grounding line retreat and readvance across Antarctica. In East Antarctica specifically, King et al. (2022)^14^ analyzed GPS rates of vertical bedrock motion and suggested late Holocene ice-sheet readvances in the Mac. Robertson Land region of East Antarctica. Additional research on mid-Holocene dynamics in West Antarctica^15–18^ has revealed similar patterns of retreat and readvance. These findings indicate a highly dynamic AIS that is capable of rapid changes and exhibits complex responses to climatic forcing. The observed patterns of retreat and readvance highlight the importance of understanding long-term ice-sheet behavior if we are to accurately predict future changes under ongoing climate warming. Furthermore, they emphasize the need for high-resolution, regionally focused studies to capture the full complexity of AIS dynamics.

Global Navigation Satellite System (GNSS) observations are a powerful tool for investigating ice-sheet fluctuations and glacial isostatic adjustment (GIA) in Antarctica. By measuring the vertical and horizontal motion of Earth’s crust, GNSS data can provide insights into the response of the solid Earth to past and present changes in ice mass^19–21^. Ohzono et al. (2006)^22^ analyzed the first GNSS observations along the Lützow–Holmbukta coast and demonstrated the potential of this technique in measuring crustal deformation and constraining GIA models. Hattori et al. (2021)^23^ analyzed GNSS data from the same region to estimate the current rate of crustal deformation and separate the GIA signal by removing the elastic deformation component related to present-day changes in ice mass. Their results further highlighted the importance of understanding the complex interplay between ice-sheet fluctuations and solid Earth processes in the Lützow–Holmbukta region.

While previous studies often use the term “readvance” to describe post-retreat ice sheet changes, our study focuses specifically on terrestrial ice-sheet “re-thickening”, which can be directly constrained by our GIA modeling approach. This distinction is important because changes in terrestrial ice thickness do not necessarily imply a significant advance in the marine grounding line, but can result from ice flow reorganization or variations in accumulation patterns. The present study aimed to build on the findings of Kawamata et al. (2020)^1^ and Suganuma et al. (2022)^2^ by integrating their geomorphological evidence and surface-exposure age data with GNSS observations^23^ to investigate the mid-Holocene history of the East Antarctic Ice Sheet (EAIS) in the Lützow–Holmbukta region. By developing models of the ice-loading history that incorporate the rapid ice-sheet thinning documented in the Skarvsnes area, and exploring scenarios that include potential re-thickening following this retreat, we consider the ice-sheet history scenarios that are most consistent with the observed crustal deformation patterns in this region.

This integrated approach allows us to: (1) constrain the mantle viscosity structure beneath East Antarctica, which is critical for understanding both past and future ice-sheet dynamics; (2) test whether GIA modeling supports the rapid mid-Holocene thinning documented at this location; and (3) examine whether the observed crustal deformation patterns are consistent with scenarios that include modest ice-sheet re-thickening following the major retreat phase. These findings contribute to the growing body of evidence regarding complex AIS fluctuations during the mid-Holocene and their potential implications for future sea-level change.

**Fig. 1:**
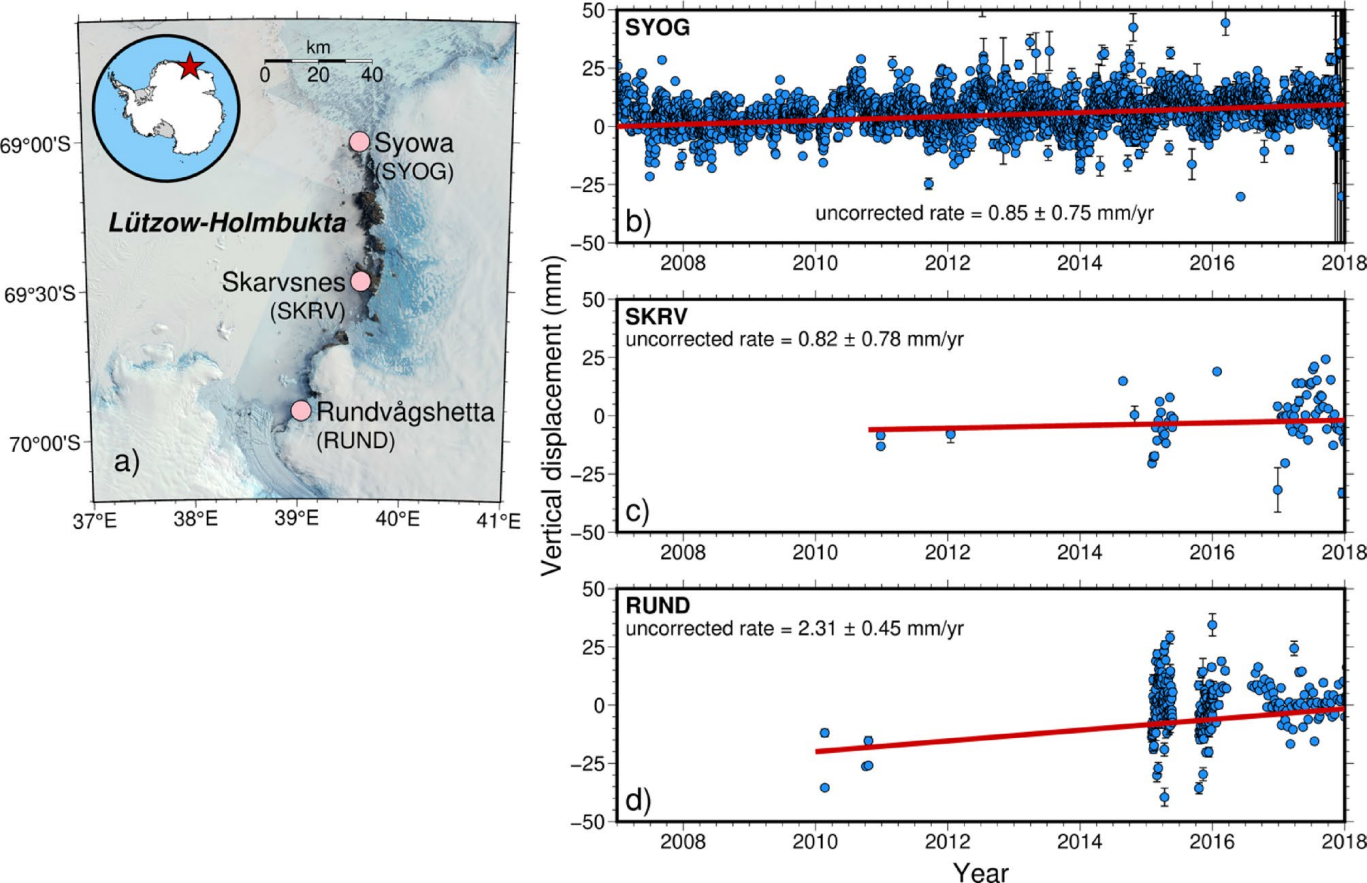
**(a)** Locations of the three Global Navigation Satellite System (GNSS) sites along the Lützow–Holmbukta coast, East Antarctica. **(b–d)** Vertical displacement time series at the three GNSS sites analyzed by Hattori et al. (2021)^23^. The vertical displacement represents the difference from the average height during the observation period at each site (upward is positive), and error bars indicate the estimated errors of the PPP analysis. The analysis periods are 2007–2018 for SYOG (Syowa Station) and 2010–2018 for the other sites. The solid red lines show the linear trends of the regressions. Maps created using GMT version 6.5.0 (https://www.generic-mapping-tools.org/). Background imagery from Landsat Image Mosaic of Antarctica (LIMA), courtesy of the U.S. Geological Survey.

## Observations

### GNSS observations and correction for elastic deformation

We selected the continuous GNSS observations from three sites along the Lützow–Holmbukta coast in East Antarctica (Fig. 1a). From north to south, these sites are the International GNSS Service (IGS) site at Syowa Station (SYOG) and two rock outcrops at Skarvsnes (SKRV) and Rundvågshetta (RUND). The raw GNSS data for each site are presented in Fig. 1b–d, along with the observed vertical crustal deformation rates derived using the data processing of Hattori et al. (2021)^23^. These data show a trend of crustal uplift since 2010. Of note, our GNSS dataset has temporal limitations. While the SYOG site has continuous observations spanning 2007–2018, the SKRV and RUND sites have more limited time series (Fig. 1b-d), primarily from observations in the early 2010s. These shorter time series potentially introduce greater uncertainty into the estimated rates of vertical crustal deformation. We have incorporated these uncertainties into our error analysis (Table 1), but acknowledge that longer observation periods would provide more robust constraints on the long-term crustal motion at these sites.

**Table 1:** Summary of GNSS observations from Hattori et al. (2021)^23^ for the Lützow–Holmbukta coast, East Antarctica.

	Coordinate (degrees)		GNSS (mm/yr)		Elastic component (mm/yr)	GIA component (mm/yr)	
Site	Latitude (°S)	Longitude (°E)	Vertical (upward, positive)	Error	GRACE	Corrected rate from GRACE	Error
Syowa station (SYOG)	69.0070	39.5837	0.85	0.75	−1.48	2.36	0.74
Skarvsnes (SKRV)	69.4738	39.6071	0.82	0.78	−1.79	2.30	0.78
Rundvågshetta (RUND)	69.9076	39.0399	2.31	0.45	−1.86	4.05	0.45

Notes: The GNSS data are the vertical linear trends and estimated errors, which were determined by the least-squares method based on the PPP solutions at each site. The corrected rate represents the GIA component after removing the elastic deformation response to present-day ice mass changes estimated from GRACE data. Abbreviations: GIA = glacial isostatic adjustment; GNSS = Global Navigation Satellite System; PPP = precise point positioning.

The elastic deformation component due to present-day changes in ice mass was estimated and removed^23^ to separate the GIA signal from the observed rates of vertical crustal deformation. We used mass-change data from the Gravity Recovery and Climate Experiment (GRACE) satellite mission to calculate the elastic deformation at each GNSS site. The elastic deformation rates are presented with the original GNSS data for the three sites in Table 1. We subtracted these elastic deformation rates from the observed rates of vertical crustal deformation to obtain the GIA component of the deformation signal (Table 1). We acknowledge the limitations of GRACE-derived elastic corrections. The spatial resolution of GRACE is ~ 300 km, which exceeds the distance between our study sites (~ 100 km apart). This results in similar elastic correction values for the three sites (Table 1). However, it is important to note that while the primary source of uncertainty lies in the GRACE mass-change estimates, the elastic response calculation itself is theoretically well-constrained using Farrell’s Green function method^24^. Additionally, Hattori et al. (2021)^23^ validated these elastic corrections through cross-comparison with ICESat satellite data, finding good agreement between the elastic responses calculated from both independent data sources. Our approach incorporates these uncertainties into the final GIA estimates using standard error propagation methods ($\:{\sigma\:}_{GIA}=\sqrt{{\sigma\:}_{GNSS}^{2}+{\sigma\:}_{elastic}^{2}}\:$), rather than assuming error-free elastic rates. These propagated uncertainties in the final GIA component estimates directly influence the plausible range of Earth rheological parameters in our analysis by defining the observational constraints used in our viscosity structure search.

**Fig. 2:**
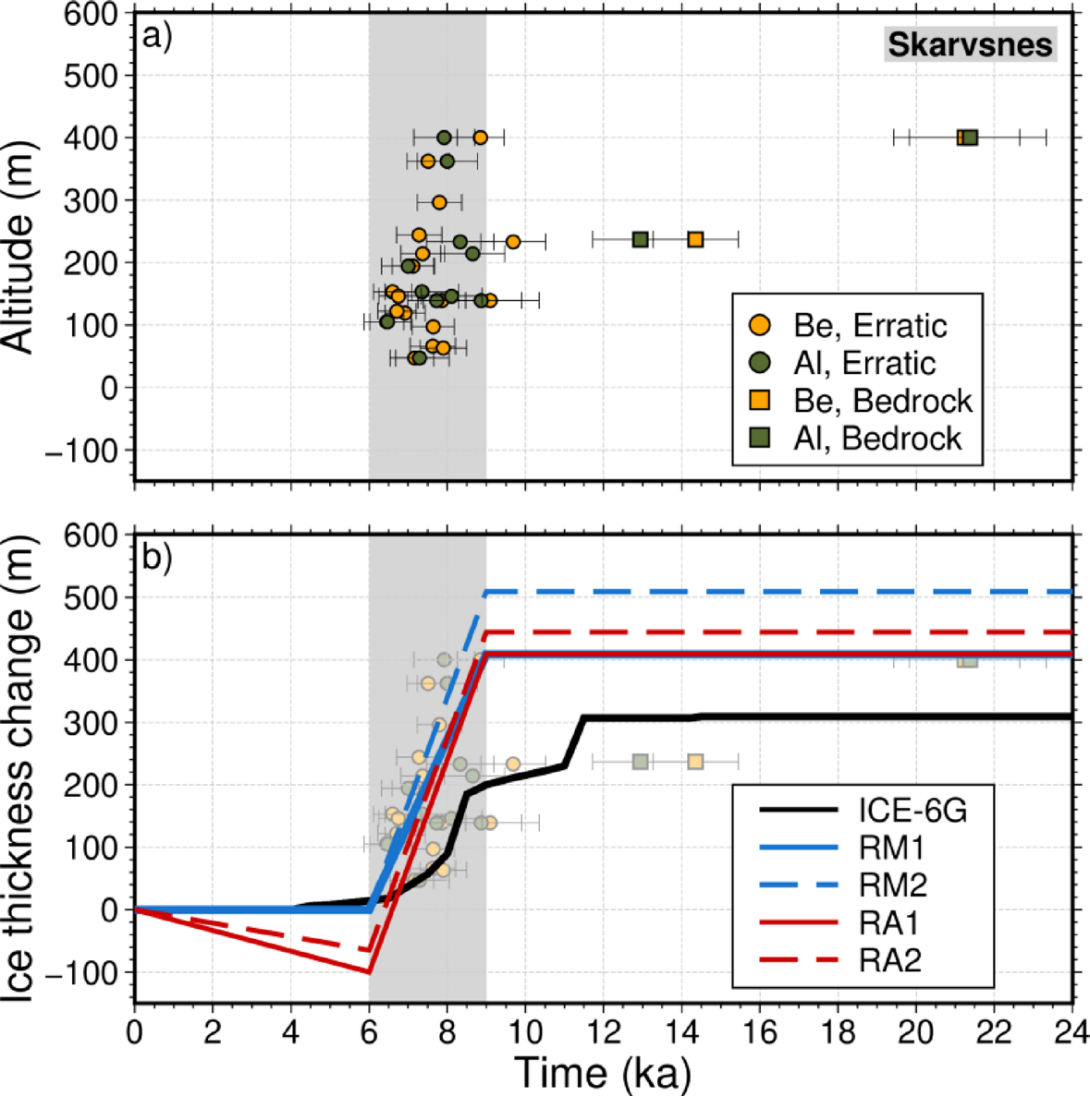
**(a)** Surface-exposure ages plotted against altitude for the Skarvsnes area (see Fig. 1a for location), modified from Kawamata et al. (2020)^1^. These data were obtained by measuring the cosmogenic^10^Be and^26^Al in bedrock outcrops and erratics in glacial landforms, which record the past ice-sheet margin. By measuring the concentrations of these nuclides, we can determine how long the surface has been exposed, providing information on the timing of glacial retreat. **(b)** Ice thickness curves across the Skarvsnes area based on the ICE-6G (black), rapid melting (RM1 and RM2), and rapid melting followed by re-thickening (RA1 and RA2) models, overlain by the surface-exposure ages shown in (**a**). The shaded gray areas in (**a**) and (**b**) denote the timing of the rapid ice-sheet thinning event (9 to 6 ka). The area of local fluctuations in ice-sheet thickness in the RM1, RM2, RA1, and RA2 models is shown in the Supporting Information as the region spanning 30–48°E and 73.5–67.5°S (Fig. 3).

### Surface-exposure dating and reconstruction of ice sheet thickness at Skarvsnes

Surface-exposure ages^1^ from the Skarvsnes area are shown in Fig. 2a. These ages reveal about 400 m of rapid ice-sheet thinning between 9 and 6 ka. We compared the surface-exposure age data with the original ICE-6G model^25^ to investigate further the changes in ice-sheet thickness at Skarvsnes (Fig. 2b). However, the ICE-6G model, which is shown as a black line in Fig. 2b, does not capture the rapid thinning that is inferred from the exposure age data.

**Fig. 3:**
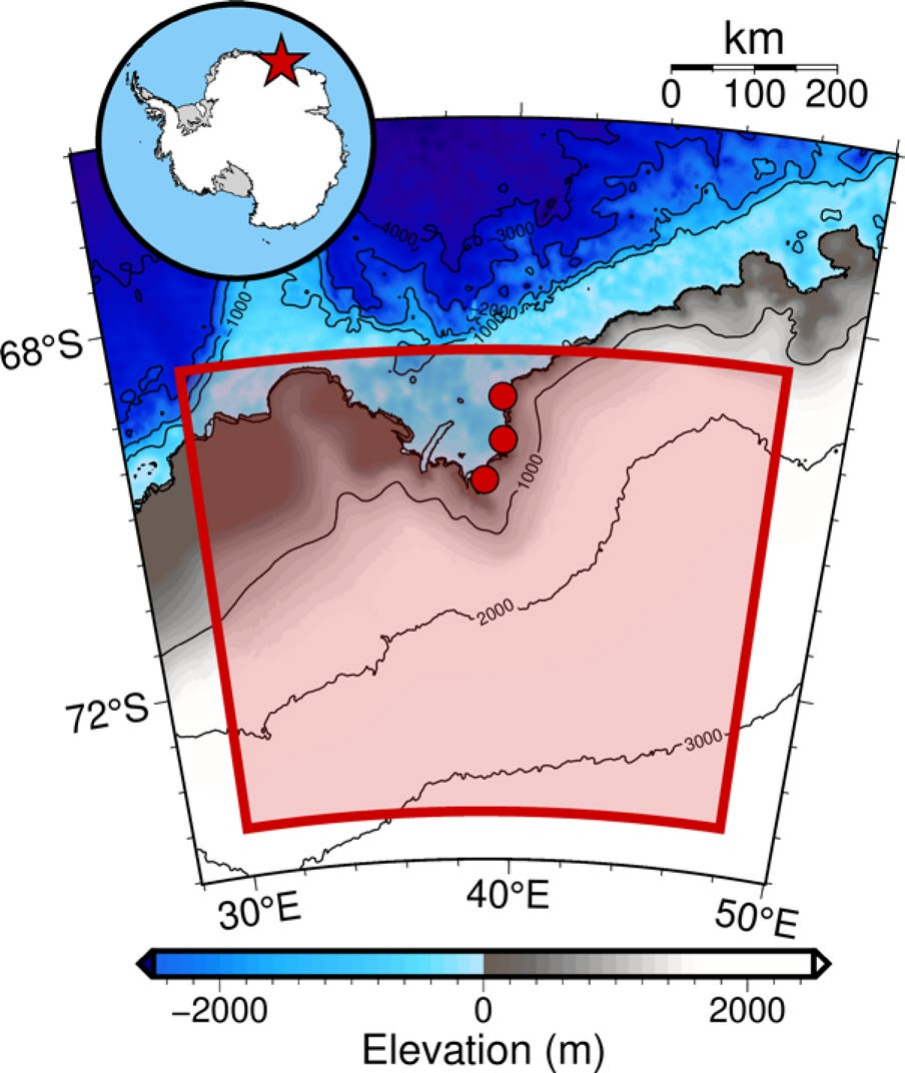
Map of the study area showing the Lützow–Holmbukta region, East Antarctica. The red polygon delineates the area where the ice-loading history was modified to capture the rapid ice-sheet thinning during the mid-Holocene, as constrained by surface-exposure ages from the Skarvsnes area^1^. Color scale and contour lines represent the topography of the ocean and land areas, respectively, and red circles denote the locations of the GNSS sites used in this study: Syowa Station (SYOG), Skarvsnes (SKRV), and Rundvågshetta (RUND). Maps created using GMT version 6.5.0 (https://www.generic-mapping-tools.org/). Topography/bathymetry from ETOPO1 Global Relief Model, courtesy of NOAA National Geophysical Data Center.

We developed a series of ice-sheet thickness reconstruction curves to better represent the fluctuations in ice-sheet thickness at Skarvsnes (Fig. 2b). The RM1 and RM2 models in Fig. 2b, which were designed to fit the surface-exposure ages^1^, show a detailed record of ice-sheet thinning in the region. We also explored scenarios (i.e., RA1 and RA2) in which the ice sheet thickened by 65–100 m following the rapid thinning event. These thickening models were included to investigate the potential for a more complex ice-sheet history in the region and assess the sensitivity of our GIA models to different histories of ice loading. The area of these local changes in ice-sheet thickness is the region of 30–48°E and 73.5–67.5°S, as shown in Fig. 3.

## Results and discussion

### Conventional and mid-Holocene rapid melting ice model

**Fig. 4:**
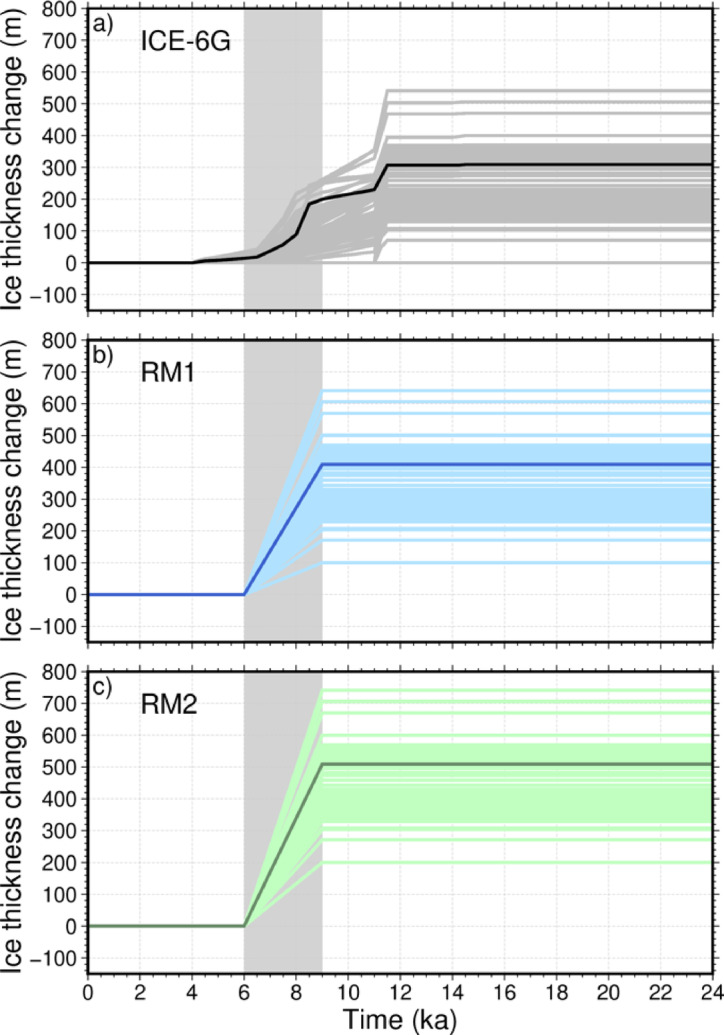
Time dependence of the ice thickness change used in this study. Darker lines in all figures indicate the ice thickness change at Skarvsnes in each model. Light lines in all figures indicate the ice thickness change at all 1,533 grid points (0.25° resolution) in the region enclosed by the red polygon in Fig. 3. **(a)** ICE-6G; **(b–c)** RM1 and RM2 only include the rapid thinning, with magnitudes of 400 and 500 m, respectively. The shaded gray areas indicate the period of rapid ice-sheet thinning from 9 to 6 ka.

Figure 3 shows the study area in East Antarctica, which focused on the Lützow–Holmbukta region (30–48°E and 73.5–68.5°S). This area is of interest due to its complex glacial history and potential for rapid changes in ice-sheet thickness. Figure 4 presents a comparative time series of ice thickness changes for three ice-sheet models: the original ICE-6G model (Fig. 4a) and our two refined models, RM1 and RM2 (Fig. 4b–c). RM1 and RM2 incorporate localized melting scenarios in the Skarvsnes area, assuming 400 m (RM1) and 500 m (RM2) of thinning, respectively, over 3 kyr from 9 to 6 ka. In Fig. 4, lighter-colored lines represent ice thickness changes for all 1,533 grid points (0.25° resolution) in the region shown in Fig. 3, while darker lines show changes specifically over Skarvsnes. This visualization highlights both regional and localized changes in the Skarvsnes area.

In developing our refined models (RM1 and RM2), we modified the ice thickness history from the ICE-6G model by incorporating local geological evidence. As shown in Fig. 4, we introduced a period of localized ice loss in the Skarvsnes area between 9 and 6 ka, with maximum thickness reductions of 400 m in RM1 and 500 m in RM2. These modifications to the ice-sheet history enable us to test whether incorporating locally constrained, rapid changes in ice-sheet thickness improves the fit between the model predictions and observations in areas with complex coastal geometries, such as Lützow–Holmbukta. In the following sections, we examine how these prescribed ice thickness histories, combined with variations in Earth’s internal viscosity structure, affect predictions of crustal deformation rates in this region.

**Fig. 5:**
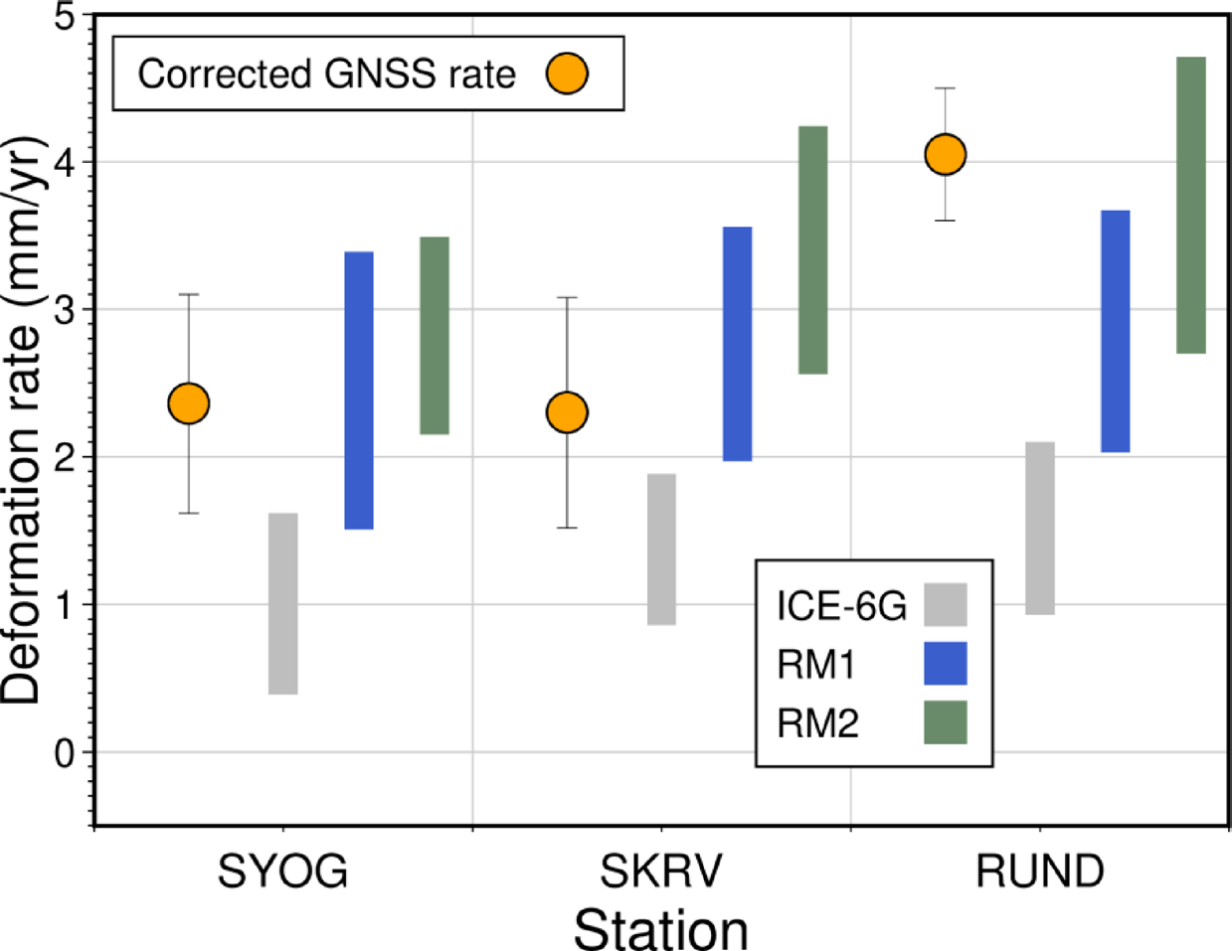
Observed GIA crustal motion at each site (orange circles) corrected for the elastic component based on the current mass change derived from GRACE^23^ (upward is positive). The gray, blue, and green bands show the full range of modeled vertical deformation rates at each GNSS site across all tested viscosity structure combinations (432 models) for the original ICE-6G^25^, RM1, and RM2 ice-loading scenarios, respectively. The colored bands encompass all possible GIA predictions within the explored parameter space of lithospheric thickness and mantle viscosities (see Fig. S1).

Figure 5 shows the GIA component of the crustal motion derived from GNSS observations^23^ and GIA model predictions using each ice model and all the viscosity models described in the previous section. Our GIA modeling results demonstrate that the reproduced deformation rates based on the original ICE-6G deglaciation history^25^ are incompatible with the GIA component of crustal motion in the Lützow–Holmbukta region^23^. However, we were able to improve the fit to the GNSS data by modifying the ICE-6G model to incorporate the rapid thinning event between 9 and 6 ka, which is indicated by the surface-exposure ages at Skarvsnes^1^ (Fig. 2). This finding highlights the importance of considering regional ice-sheet histories when modeling Antarctic GIA, as the local deglaciation pattern can differ markedly from the continental-scale trends that are captured by global models such as ICE-6G. To quantitatively assess these visual improvements, we calculated weighted root mean square (WRMS) misfits between observed and predicted deformation rates. The ICE-6G model shows poor quantitative fit with WRMS = 2.65 mm/yr. In contrast, models incorporating rapid mid-Holocene thinning show substantial improvements: RM1 (WRMS = 0.89 mm/yr, 66% improvement) and RM2 (WRMS = 0.85 mm/yr, 68% improvement). *F*-tests confirm these represent substantial, though not statistically significant, improvements over ICE-6G (RM1: *p* = 0.058; RM2: *p* = 0.052) shown in Table 2. While these results suggest that the RM1 and RM2 models can adequately reproduce the observed vertical uplift rates at each GNSS site, we conducted a more detailed analysis to examine how these predicted rates at individual sites respond to variations in mantle viscosity structure.

**Fig. 6:**
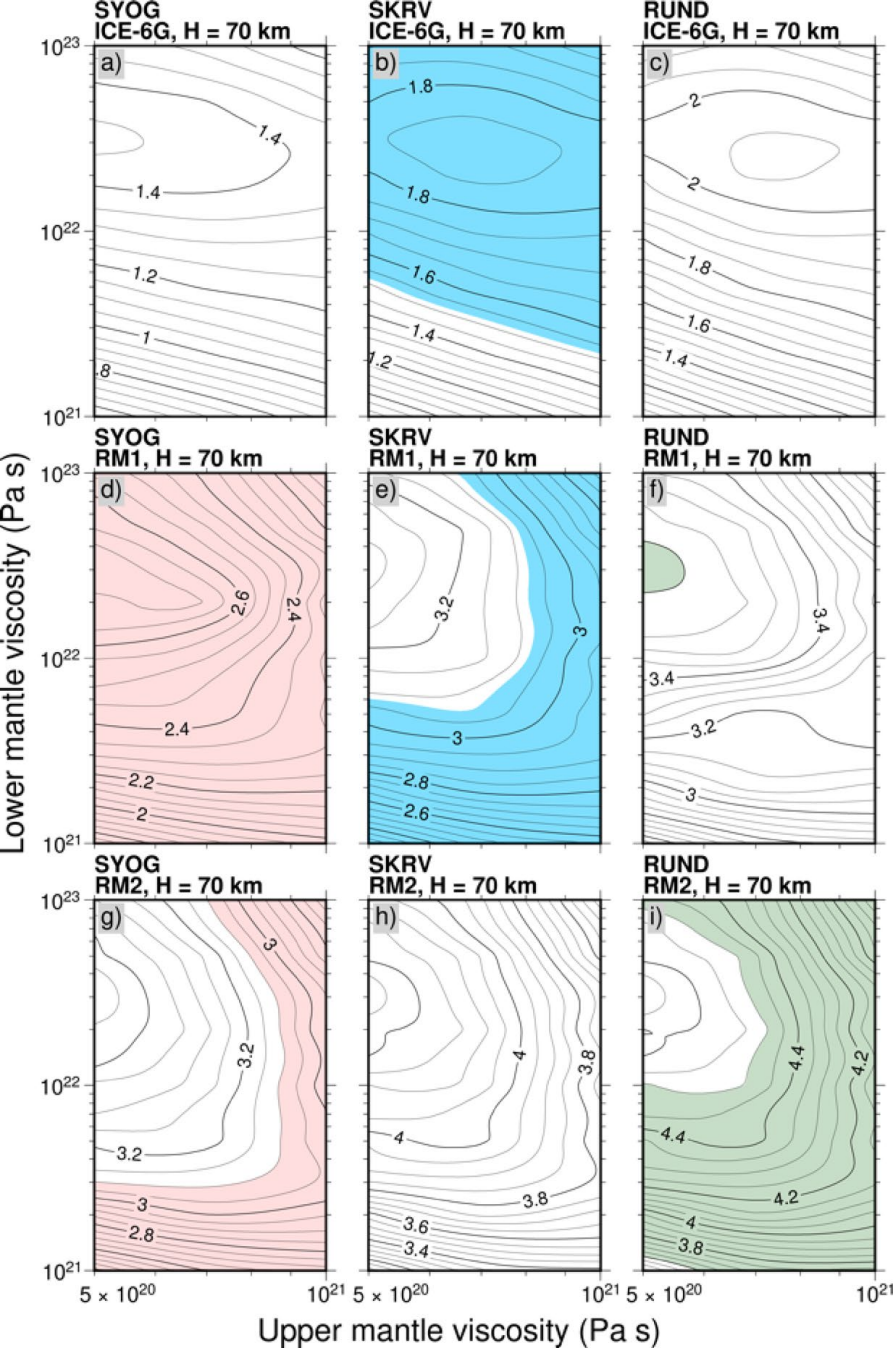
Predicted rates of crustal deformation at each GNSS site as a function of the upper- and lower-mantle viscosities. The lithospheric thickness (*H*), whereby the top layer behaves as the elastic layer, is 70 km for all the models depicted here; the results of other elastic thickness models are shown in Figs. S2–S3. The color-shaded regions indicate the permissible ranges constrained by the GNSS observations at each site. ICE-6G, RM1, and RM2 were adopted as the ice-sheet history models.

We conducted a precise viscosity analysis that was dependent on the predicted deformation rates, with the results shown in Fig. 6. We calculated the predictions at each site based on the ICE-6G, RM1, and RM2 ice-sheet history models and all the viscosity models, with the color-shaded regions highlighting the permissible range of the GIA component of the deformation rates derived from the GNSS observations^23^ (Table 1). The viscosity models span a range of upper-mantle viscosities, from 5 × 10^20^ to 1 × 10^21^ Pa s, and lower-mantle viscosities, from 1 × 10^21^ to 1 × 10^23^ Pa s, allowing us to explore a comprehensive parameter space of possible Earth rheological structures, which are shown in Fig. S1 as a radial viscosity profile. Figure 6 presents the results for a fixed lithospheric thickness of 70 km for all three ice models (ICE-6G, RM1, and RM2). Additional calculations with various lithospheric thickness assumptions are provided in the Supplementary Figures (Figs S2–S3). For each observation site, we systematically compared the predicted uplift rates with the observed GIA components, paying particular attention to the sensitivity of the predictions to variations in upper- and lower-mantle viscosities. The analysis revealed distinct patterns of model prediction sensitivity at different sites, with some locations showing a strong dependence on upper-mantle viscosity, and others exhibiting more complex responses involving the properties of both the upper and lower mantle. Figure 6 shows there is no unique solution to the viscosity structure that explains the GNSS observations at all three sites, thereby suggesting that a simple model of mid-Holocene rapid melting is insufficient for reproducing the observed crustal deformation patterns based on the GIA modeling. This result indicates that either the spatial pattern of ice loss during the mid-Holocene was more complex than our current models suggest or that regional variations in Earth’s rheological properties have a stronger influence on local crustal deformation rates than previously assumed^8,26,27^.

### Ice-sheet re-thickening model

The above results suggest that the mid-Holocene rapid thinning alone may not completely explain the observed GIA patterns in the Lützow–Holmbukta region. Recent studies have provided compelling evidence for a more complex ice-sheet evolution than scenarios considering only ice-sheet retreat during the Holocene, particularly regarding post-thinning stabilization or modest readvance of the ice sheet. Notably, Bradley et al. (2015)^11^ reported evidence of late Holocene ice-sheet readvance in the Weddell Sea region based on low rates of post-glacial rebound, while Kingslake et al. (2018)^12^ identified extensive retreat and subsequent readvance of the West Antarctic Ice Sheet during the Holocene. In East Antarctica, geological data along the coastal Lützow–Holmbukta region^28^ and glaciological evidence in the interior^29^ indicate ice-sheet fluctuations after the mid-Holocene thinning. Motivated by these findings, we developed additional loading scenarios that incorporate both the rapid mid-Holocene thinning and subsequent variations in ice-sheet thickness. These new scenarios aimed to test whether a more complex loading history, including potential ice-sheet re-thickening could better explain the observed present-day patterns of crustal deformation in the study area.

**Fig. 7:**
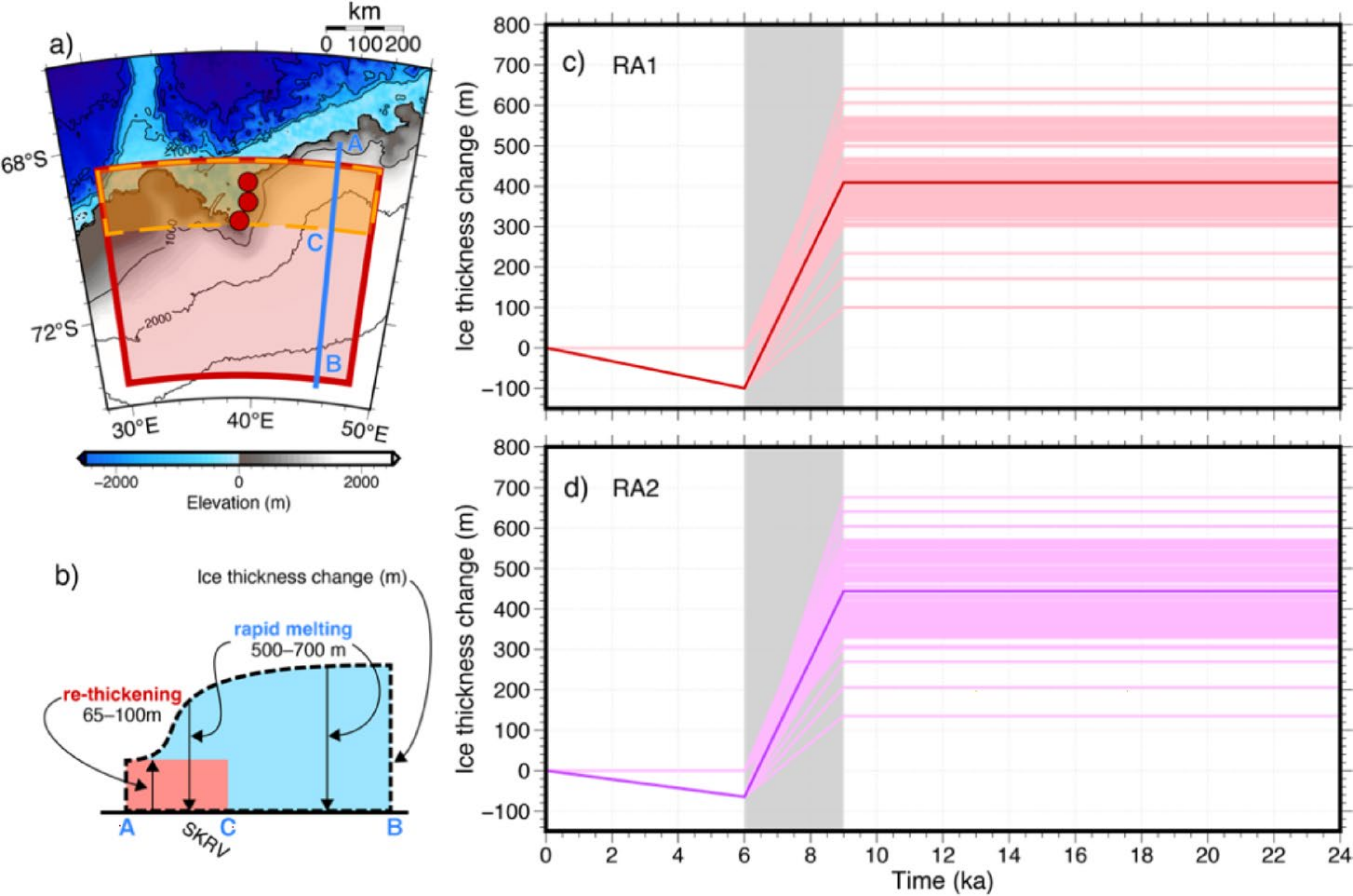
**(a)** Region of ice-sheet re-thickening (region bounded by the dashed orange line) considered in the RA1 and RA2 models, which is overlaid on the same map as in Fig. 3, where the area of rapid mid-Holocene thinning (region bounded by the red line) in the Lützow–Holmbukta region is shown. **(b)** Schematic cross-section illustrating the rapid melting during the Holocene and subsequent re-thickening. This panel represents a simplified view of the area indicated by the blue line in (**a**). Note that the absolute thickness of the ice sheet is not shown, and only relative changes are depicted. **(c–d)** Time series of ice-sheet thickness modeled to represent the re-thickening phase (RA1 and RA2, respectively). The bold lines in each panel indicate changes at the Skarvsnes site, where surface-exposure age data have been obtained. The thin lines represent ice-sheet thickness for all grid points in the area indicated in (**a**). In both scenarios, the rapid Holocene melting is assumed to be 500 m. The subsequent re-thickening is modeled as 100 m in RA1 (**c**) and 65 m in RA2 (**d**). Map created using GMT version 6.5.0 (https://www.generic-mapping-tools.org/). Topography/bathymetry from ETOPO1 Global Relief Model, courtesy of NOAA National Geophysical Data Center.

To investigate the effects of a possible ice-sheet re-thickening following the mid-Holocene rapid thinning, we considered additional ice-loading scenarios for the Lützow–Holmbukta region (Fig. 7). Figure 7a shows the spatial extent of both the region of rapid thinning (identical to Fig. 3) and the restricted area of subsequent re-thickening (area bounded by the dashed orange line) over 68.5–70°S for a consistent longitudinal extent (30–48°E). Figure 7b presents a schematic N–S cross-section showing that the ice sheet re-thickening was limited to the area bounded by the dashed orange line, while the surrounding region maintained its thinned state after the initial rapid melting between 9 and 6 ka. The spatial relationship between the rapidly thinned and re-thickened areas suggests a localized response to regional climate or glaciological factors. The temporal evolution of ice thickness under these new scenarios is presented in Fig. 7c–d, following the same format as in Fig. 4. These models, designated RA1 and RA2, maintain the initial 400 m of rapid thinning between 9 and 6 ka, but incorporate subsequent ice-sheet variations. Figure 7c shows the RA1 scenario with a 100 m increase in ice thickness during the re-thickening phase, while Fig. 7d presents the RA2 scenario with a relatively moderate 65 m increase in ice thickness. Note that the different spatial extent of the re-thickening area relative to the initial thinning region results in non-proportional differences between these scenarios. The models that incorporate these 65–100 m ice re-thickening events after the rapid thinning are consistent with the surface-exposure age data and provide an alternative interpretation to models without subsequent re-thickening (see Fig. 2). This finding suggests that the EAIS in the Lützow–Holmbukta region may have experienced a more complex deglaciation history than previously thought, potentially characterized by periods of rapid retreat followed by intervals of ice-sheet stability and/or modest re-thickening.

**Table 2:** Statistical comparison of ice load models based on fit between predicted and observed GIA uplift rates.

Ice load model	WRMS (mm/yr)	χ^2^	Improvement	*F-*value	*p*-value	Significance	Station Predictions (mm/yr)		
							SYOG	SKAR	RUND
ICE-6G	2.65	21.02	–	–	–	–	1.29	1.87	2.11
RM1	0.89	2.38	66.4%	15.67	0.058	ns	2.44	3.27	3.64
RM2	0.85	2.14	68.1%	17.62	0.052	ns	2.34	3.26	3.70
RA1	0.69	1.45	73.8%	27.07	0.035	*	2.09	3.04	3.76
RA2	0.72	1.54	72.6%	24.28	0.038	*	2.16	3.11	3.77

WRMS represents weighted root-mean-square residuals between observed and predicted GIA rates. *F*-value measures how much better each model fits the data compared to ICE-6G (higher *F*-value = greater improvement). *p*-value indicates the statistical significance of this improvement (lower *p*-value = more confident that the improvement is real, not due to chance). Station predictions show model-predicted uplift rates at the optimal viscosity structure for each ice model. *: statistically significant improvement (*p* < 0.05); ns: not significant.

**Fig. 8:**
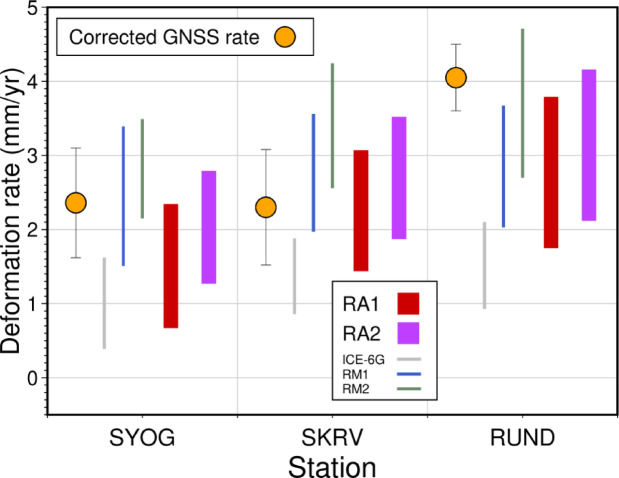
Observed GIA crustal motion at each site (orange circles) corrected for the elastic component based on the current mass change derived from GRACE^23^ (upward is positive). The red and purple bands show the full range of modeled vertical deformation rates across all tested viscosity structure combinations for the re-thickening scenarios (RA1 and RA2). The bands represent the complete envelope of GIA predictions spanning the explored rheological parameter space. Results for the ICE-6G, RM1, and RM2 models are also shown as thin bands for comparison.

Figure 8 shows the predicted rates of crustal deformation for the RA1 and RA2 models across the full range of tested mantle viscosity structures at each observation site, following the same presentation format as in Fig. 5. While these new models that incorporate post-thinning variations in ice-sheet thickness show slightly larger deviations from the observed values as compared with the models of rapid thinning alone, they still provide substantially better agreement with the GNSS observations than the original ICE-6G model. The predicted ranges of deformation rates, encompassing all possible viscosity structures, fall mainly within the observational error bounds at each site. This suggests that the inclusion of moderate ice-sheet re-thickening (65–100 m) following the rapid thinning event is consistent with the observed GIA signals and yields a more comprehensive representation of the ice-sheet history in this region than the original ICE-6G model. To evaluate these improvements quantitatively, we calculated statistical measures for model performance (Table 2). The analysis confirms the superior performance of re-thickening models: RA1 (WRMS = 0.69 mm/yr) and RA2 (WRMS = 0.72 mm/yr) achieve 74% and 73% improvements over ICE-6G, respectively. Importantly, *F*-tests demonstrate these re-thickening models provide statistically significant improvements over ICE-6G (RA1: *p* = 0.035; RA2: *p* = 0.037), supporting the hypothesis that modest re-thickening followed the rapid mid-Holocene thinning event.

**Fig. 9:**
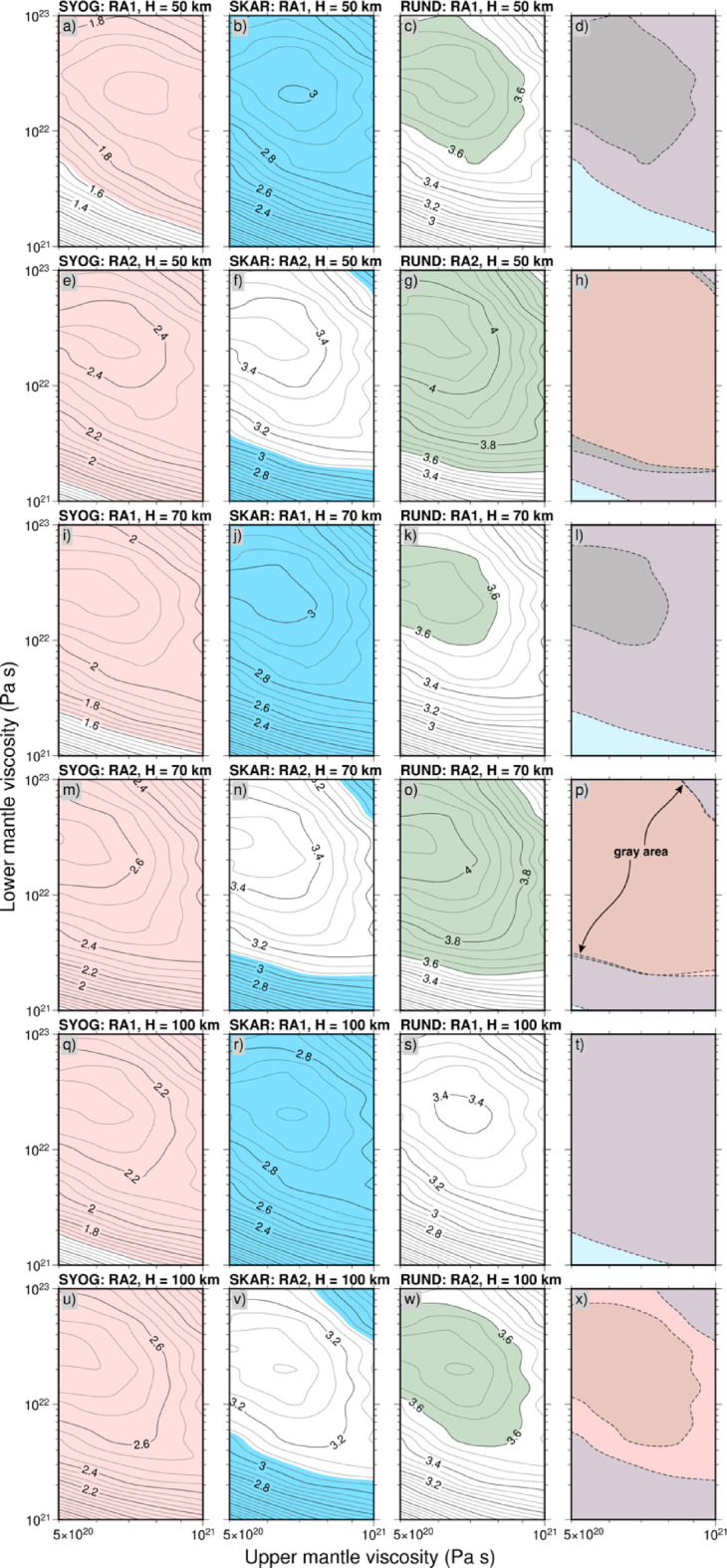
Predicted rates of crustal deformation at each site as a function of upper- and lower-mantle viscosities. Adopted lithospheric thicknesses of **(a–h)** 50 km, **(i–p)** 70 km, and **(q–x)** 100 km are shown, and the results for other models of elastic thickness are shown in Fig. S4. The color-shaded regions indicate the permissible ranges constrained by the GNSS observations at each site. RA1 and RA2 were adopted here as the models of ice-sheet history. The gray areas in (d), (h), (i), and (p) indicate the parts of the viscosity range that are satisfied by the individual solutions from the GNSS observations at each site. (t) and (x) have no solutions that satisfy the observations at each site.

Figure 9 presents a detailed analysis of the prediction results using the RA1 and RA2 ice-sheet history models, focusing on the dependence of the predictions on lithospheric thickness and mantle viscosity structure. Our analysis revealed two key findings. First, we identified viscosity solutions that can adequately explain the GNSS observations at all three sites when assuming lithospheric thicknesses of 50 and 70 km. However, with a lithospheric thickness of 100 km, there is no viable solution that can simultaneously satisfy the observational constraints at all sites. More specifically, a lithospheric thickness of 50–70 km combined with an upper-mantle viscosity of 5–7 × 10^20^ Pa s provided the best fit to the observed rates of surface deformation, with other elastic thickness model results shown in Figs. S4 and S5. Second, our analysis revealed two distinct groups of viable mantle viscosity solutions: (1) models characterized by a lower-mantle viscosity of 2–3 × 10^21^ Pa s, with little sensitivity to upper-mantle viscosity; and (2) models requiring both an upper-mantle viscosity below 8 × 10^20^ Pa s and a highly viscous lower mantle (> 10^22^ Pa s). These solution groups exhibit a systematic relationship with the magnitude of ice-sheet re-thickening. Solutions incorporating a higher viscosity in the lower mantle are preferentially associated with scenarios of greater re-thickening, potentially reflecting a mechanism whereby the increased viscosity moderates the enhanced deformation rates induced by larger loading variations. In contrast, scenarios with moderate re-thickening are more consistently explained by solutions with a lower-viscosity structure that effectively responds to smaller-amplitude loading changes while maintaining compatibility with the observed deformation rates. The models incorporating moderate ice-sheet thickening (65–100 m) after the rapid thinning event not only provide these specific viscosity solutions, but also show improved consistency with local geological constraints, particularly the surface-exposure dating results. These findings point to a more nuanced understanding of the Holocene evolution of the EAIS in the Lützow–Holmbukta region, consistent with a complex deglaciation history that may have included distinct phases of retreat followed by periods of stabilization or modest re-thickening. While our data and modeling do not definitively prove ice-sheet re-thickening occurred, they are compatible with scenarios that include such a phase following the major mid-Holocene retreat. The progressive improvement in model fit achieved by RM models over ICE-6G, and subsequently by RA models over both ICE-6G and RM models, confirmed through WRMS reduction and statistical significance testing (Table 2), demonstrates the importance of incorporating regionally-constrained ice histories when investigating past ice-sheet dynamics. The quantitative analysis supports the occurrence of post-thinning ice-sheet stabilization or modest re-thickening in the Lützow-Holmbukta region, moving beyond visual assessment to provide statistical rigor for our interpretation of complex mid-Holocene ice-sheet evolution.

**Table 3:** Optimal Earth rheological parameters from this study compared with previous estimates. Values in parentheses indicate uncertainty ranges based on models that satisfy observational constraints within error bounds. Upper and lower mantle viscosity values represent best-fit parameters from re-thickening scenarios (RA1, RA2) that show statistically significant improvements over ICE-6G.

	Lithospheric Thickness (km)	Upper Mantle Viscosity (×10^20^ Pa s)	Lower Mantle Viscosity (×10^21^ Pa s)	Study Region	Reference
This study:					
RA1	60 (50–70)	6 (5–7)	20 (6–80)	Lützow–Holmbukta	–
RA2	50 (50–70)	5 (5–10)	5 (3–5, 50–100)	Lützow–Holmbukta	–
Previous studies:					
GIA model	50–100	1	20	Antarctica	Whitehouse et al. (2012)^8^
GIA model	70–120	2–10	10–100	Antarctica	Ivins and James (2005)^26^
Gravity data	60–80			East Antarctica	Chen et al. (2018)^30^

Lithosphere thickness represents the elastic layer thickness in GIA modeling. Upper mantle viscosity spans from the base of the lithosphere to 670 km depth. Lower mantle viscosity represents the average viscosity from 670 km to the core-mantle boundary. Uncertainty ranges for this study reflect the full parameter space of models achieving a good fit to GNSS observations. Previous studies used different observational constraints: relative sea-level data (Whitehouse et al. 2012^8^), GPS data in Antarctica (Ivins and James 2005^26^), and gravity data (Chen et al. 2018^30^).

### Constraints on Earth’s rheological structure

The sensitivity of our GIA model predictions to the adopted viscoelastic Earth structure is clearly evident in Figs. 8 and 9, as well as Fig. S3. Our analysis identified optimal solutions with lithospheric thickness of 50–70 km and upper-mantle viscosity of 5–7 × 10^20^ Pa s that provided the best fit to the observed rates of surface deformation (Table 3). Our preferred lithospheric thickness of 50–70 km shows excellent agreement with the effective elastic thickness (*Te*) determined for East Antarctica through detailed gravity and topography analysis^30^, where *Te* is treated as equivalent to the lithospheric thickness in this study. Chen et al. (2018)^30^ reported *Te* = 60–80 km for most cratonic regions, providing compelling independent validation through completely different observational approaches (gravity-topography spectral analysis versus geodetic GIA modeling). Their comprehensive analysis revealed regional variations, with the Aurora Subglacial Basin showing the highest values (~ 90 km) and the Lambert Graben showing anomalously low values (~ 15 km), demonstrating the expected variability in lithospheric properties across East Antarctica. This convergence highlights the complementary value of different geophysical methods in constraining Earth’s lithospheric properties. Our estimates are consistent with the lower end of ranges reported by Whitehouse et al. (2012)^8^ and Ivins and James (2005)^26^ shown in Table 3, while showing broad compatibility with seismological studies across Antarctica^31–33^. However, our values represent the thinner lithospheric regime characteristic of coastal East Antarctica, in contrast to the thick cratonic lithosphere (150–250 km) documented in the continental interior by An et al. (2015)^31^, reflecting the expected regional variation from coast to craton.

Our upper mantle viscosity estimates (5–7 × 10^20^ Pa s) are significantly higher than the 1 × 10^20^ Pa s reported by Whitehouse et al. (2012)^8^, though they fall within the broader range suggested by Ivins and James (2005)^26^. Our lower-mantle viscosity estimates (5–80 × 10^21^ Pa s) are consistent with well-established global viscosity models^34–36^, demonstrating the complementary nature of geodetic and seismological constraints on Earth’s rheological structure.

The uncertainty ranges presented in Table 3 reflect the inherent limitations of constraining Earth’s rheological structure using geodetic observations from a spatially limited network. While our analysis identified optimal viscosity solutions that satisfy observational constraints at all three GNSS sites, Fig. 9 reveals that multiple parameter combinations can provide similarly good fits to the data. This non-uniqueness of solutions is particularly evident in the lower mantle viscosity estimates. Our analysis revealed two distinct groups of viable mantle viscosity solutions: (1) models characterized by a lower-mantle viscosity of 2–3 × 10^21^ Pa s, with little sensitivity to upper-mantle viscosity; and (2) models requiring both an upper-mantle viscosity below 8 × 10^20^ Pa s and a highly viscous lower mantle (> 10^22^ Pa s).

The viscosity constraints show notable differences between re-thickening scenarios. RA1 favors moderate lower-mantle viscosities (6–80 × 10^21^ Pa s), while RA2 exhibits a distinctive bimodal distribution with solutions clustering around either low viscosities (3–5 × 10^21^ Pa s) or high viscosities (50–100 × 10^21^ Pa s). This bimodal pattern suggests that the magnitude of ice-sheet re-thickening fundamentally influences the permissible range of mantle rheological properties, with smaller re-thickening events (RA2: 65 m) allowing for more diverse viscosity structures compared to larger re-thickening scenarios (RA1: 100 m).

The broad parameter ranges that satisfy our observational constraints highlight both the sensitivity of GIA predictions to rheological structure and the challenges of uniquely constraining mantle properties from surface deformation measurements alone. The lithospheric thickness estimates show the most consistent constraint (50–70 km), likely reflecting the dominant influence of this parameter on short-wavelength deformation patterns captured by our local GNSS network. In contrast, the greater uncertainty in mantle viscosity estimates reflects the trade-offs between upper and lower mantle properties that can produce similar surface deformation signatures, particularly when constrained by observations from a geographically limited region.

These discrepancies likely reflect fundamental differences in observational constraints and methodological approaches. Whitehouse et al. (2012)^8^ used relative sea-level data spanning much longer timescales and broader spatial coverage across Antarctica, potentially sampling different rheological regimes or averaging over lateral heterogeneities. In contrast, our analysis focuses on present-day crustal motion in a geographically limited coastal region, providing constraints on contemporary viscosity structure that may differ from long-term averaged properties. Additionally, the incorporation of regionally-constrained ice-loading histories in our study (RA1 and RA2 models) may require different rheological parameters compared to studies using global deglaciation models like ICE-6G.

While our study demonstrates that a simplified three-layer viscoelastic Earth structure can explain the local GIA observations in the Lützow–Holmbukta region, it is worth considering how our approach relates to more complex three-dimensional (3D) Earth models. Comparative studies of crustal deformation rates predicted by one-dimensional (1D) and 3D Earth models across Antarctica^27,37,38^ have revealed that significant differences occur mainly in regions with pronounced lateral variations in mantle structure. In contrast, in regions with relatively homogeneous lithospheric and mantle properties (e.g., our study area in the coastal region of East Antarctica) the differences between 1D and 3D model predictions are much smaller. The Lützow–Holmbukta region is situated away from major structural boundaries and transition zones identified in seismological studies^31,33^, suggesting that lateral heterogeneities have minimal influence on GIA predictions in this specific area. Furthermore, our comprehensive exploration of 432 distinct viscosity structure combinations, which were tested against 20 ice-loading histories (of which 5 representative models are presented here), effectively captures a wide range of possible solid Earth responses. This thorough parameter space investigation, encompassing thousands of unique model scenarios, allows for a reasonable assessment of local ice-history variations, despite the inherent limitations of a 1D Earth model framework. While our systematic approach suggests that our findings regarding mid-Holocene ice-sheet dynamics, including the potential for modest re-thickening following major thinning, are adequately supported, we acknowledge that future integration of 3D Earth models may refine specific quantitative aspects of our results. With these considerations of Earth structure in mind, we now turn to the broader implications of our findings for regional ice-sheet history and dynamics.

### Implications for regional ice-sheet history and dynamics

The integrated results from our GIA modeling and existing geological evidence have important implications for our understanding of the history and dynamics of the EAIS in the Lützow–Holmbukta region. The rapid thinning event between 9 and 6 ka, as recorded by the surface-exposure age data^1^ and captured by our GIA modeling, suggests the EAIS in this region was highly sensitive to climate forcing during the mid-Holocene. This finding aligns with growing evidence for substantial ice-sheet retreat and thinning across other parts of East Antarctica during this period^1–3,9^. Similar patterns of rapid ice loss have been documented in the Wilkes Land sector^39^ and along the George V Coast^9^, suggesting a coherent response of the EAIS margin to mid-Holocene warming.

The potential for a re-thickening of the EAIS following the rapid retreat event, as suggested by our modeling results, raises important questions about the mechanisms that caused these changes. One possibility is that the initial retreat was driven by a combination of atmospheric and oceanic warming, leading to increased surface melting and enhanced basal melting of ice shelves, as suggested by Suganuma et al. (2022)^2^ and supported by marine sediment records^39^. The subsequent re-thickening may have been driven by multiple factors: a cooling climate^40^, the stabilizing effect of isostatic rebound, which would have reduced the relative sea level and promoted ice-sheet grounding^12^, and potential changes in ocean circulation patterns^41^.

Recent studies have identified similar patterns of retreat and partial recovery in other sectors of Antarctica. For example, Wise et al. (2017)^42^ reported post-retreat stabilization in the Ross Sea sector, while Small et al. (2019)^43^ found indications of ice-sheet reorganization in the Weddell Sea region. These findings suggest that the behavior observed in the Lützow–Holmbukta region may represent a more widespread pattern of ice-sheet response to mid-Holocene climate variations, rather than a purely local phenomenon.

The timing and magnitude of these changes have implications for our understanding of ice-sheet stability mechanisms. Our findings suggest that the EAIS can undergo rapid changes on timescales of centuries to millennia, but also possesses mechanisms for stabilization and partial recovery. This behavior is particularly relevant for projecting future ice-sheet responses to warming, as it indicates both the potential vulnerability of the EAIS to rapid change and its capacity for stabilization under favorable conditions^44,45^.

### Limitations and future work

While our GIA modeling approach has provided new insights into the history and dynamics of the ice sheet in the Lützow–Holmbukta region, several important limitations need to be acknowledged. First, the surface-exposure age data used to constrain the ice-loading history are subject to uncertainties related to the calibration of production rate, scaling methods, and potential inheritance or erosion^46^. These uncertainties can affect our interpretation of the timing and magnitude of ice sheet changes. Future work should aim to refine the surface-exposure age dataset and incorporate additional geological constraints, including marine sediment records and raised beach deposits, to better constrain the timing and magnitude of ice-sheet changes.

Second, while our study demonstrates that a simplified three-layer viscoelastic Earth structure can explain the local GIA observations in the Lützow–Holmbukta region, we acknowledge that this model may have limitations when applied to broader areas of Antarctica. The success of our simple three-layer model in explaining local geodetic observations suggests that, for regional-scale studies with good observational constraints, such simplified Earth models can be sufficiently robust. However, recent seismological studies^31–33^ have revealed significant lateral variations in lithospheric thickness and mantle viscosity across Antarctica, which could become crucial when extending the analysis to larger spatial scales or different regions. Furthermore, the incorporation of nonlinear rheologies, such as power-law flow or transient creep^47^, may provide additional insights when considering Antarctica-wide GIA responses, particularly during periods of rapid deglaciation.

Finally, our study focuses on a single region within East Antarctica, and our findings might not directly apply to other parts of the EAIS. Integrating geological, geodetic, and modeling constraints from multiple sites across East Antarctica is essential to obtaining a comprehensive understanding of the mid-Holocene EAIS history and its implications for future sea-level change. Such a research focus will require a coordinated effort from the glaciological, geological, and geophysical communities to collect and synthesize data from remote parts of the continent.

Despite these limitations, our study demonstrates the value of integrating surface-exposure age data, geodetic observations, and GIA modeling to constrain the history and dynamics of the EAIS in the Lützow–Holmbukta region. Our results highlight the potential for rapid ice-sheet thinning and re-thickening during the mid-Holocene, with implications for the sensitivity of the EAIS to climate forcing. By refining our understanding of the past behavior of the EAIS, we can improve our ability to predict its future response to anthropogenic climate change and the associated impacts on global sea levels.

Our integrated approach, which combines previously published surface-exposure age data, geodetic observations, and GIA modeling, provides new insight into the behavior of the EAIS in the Lützow–Holmbukta region. The GIA modeling supports the rapid ice-sheet thinning between 9 and 6 ka that was documented by Kawamata et al. (2020)^1^ and suggests scenarios that include modest re-thickening (65–100 m) are consistent with geodetic observations in this region. While we cannot definitively prove that such re-thickening occurred, our results demonstrate that GIA models incorporating this phase show improved agreement with observational constraints compared with the global ICE-6G model. The identification of optimal Earth model parameters (lithospheric thickness of 50–70 km and specific ranges of mantle viscosities) represents a significant contribution of this work (Fig. S5), as these parameters show remarkable consistency with independent constraints from seismological studies^31–33^ and previous GIA modeling^8,20,26^. This consistency validates our regional GIA modeling approach, despite the acknowledged limitations of using a 1D Earth model and the spatial and temporal constraints of our GNSS dataset. Future research should aim to extend this approach to other regions of East Antarctica by integrating additional geological and geophysical data, longer GNSS time series, and potentially 3D Earth structure models to develop a more comprehensive understanding of the mid-Holocene ice-sheet history. This expansion of our knowledge of past ice-sheet dynamics, particularly during periods of rapid change followed by potential stabilization or recovery, is crucial for improving predictions of the EAIS response to ongoing climate change. Such an understanding is essential for developing effective climate adaptation strategies and managing the risks posed by future sea-level rise to coastal communities worldwide.

## Methods

### GNSS data processing and uncertainty estimation

We analyzed continuous GNSS observations from SYOG at Syowa Station (2007–2018) and campaign observations from Skarvsnes (SKRV) and Rundvågshetta (RUND) along the Lützow–Holmbukta coast, following the established methodology of Hattori et al. (2021)^23^. These sites were selected for their stable bedrock locations and accessibility for geodetic measurements (Fig. 1a). SYOG, operated by the Geospatial Information Authority of Japan since 1995, provides continuous observations with equipment replacement conducted in 2013 for multi-GNSS capability^23^. The four outcrop sites represent campaign-style observations that gradually transitioned to automated systems during the study period, each equipped with a GEM-1 receiver connected to a JAVA GrAnt-G3T antenna, powered by lead-acid batteries and solar generation systems, with weekly 24-hour observation cycles.

However, our GNSS dataset has temporal limitations. While the SYOG site has continuous observations spanning 2007–2018, the SKRV and RUND sites have more limited time series (Fig. 1b-d), primarily from observations in the early 2010 s, potentially introducing greater uncertainty into the estimated rates of vertical crustal deformation.

Site positions were estimated using the precise point positioning (PPP) static method with the “c5++” multi-technique space geodetic analysis software^48^ and antenna model IGS14.atx. We used ionosphere-free combinations of GPS L1/L2 measurements without higher-order ionosphere corrections, with station positions estimated every 24 h and receiver clock offsets estimated every 30 s using IGS Final products for satellite orbits and clocks. The analysis included removal of solid Earth tides (semi-diurnal to 18.6 years), oceanic tidal loading using the EOT11a model^49^, and non-tidal ocean and atmospheric loading corrections using International Mass Loading Service data^50^. Tropospheric delays were corrected using the Global Mapping Function^51^. For velocity estimation, we employed different approaches for continuous and campaign sites. For the continuous SYOG station, we used Hector software^52^ with a combined flicker noise and white noise model to properly account for temporally correlated noise in GPS time series, with corrections for equipment offsets in 2013. For outcrop sites, velocities were estimated using weekly solutions and campaign observations with linear regression models, including annual and semi-annual components after removing outliers exceeding 10 cm from the median^23^. The velocity estimation incorporated both deterministic and stochastic components:

$$\:x\left({t}_{i}\right)=x_{0}+v\cdot t_{i}+a \cos\left(2\pi\frac{doy}{365.25}\right)+b \sin\left(2\pi\frac{doy}{365.25}\right)+c \cos\left(4\pi\frac{doy}{365.25}\right)+d \sin\left(4\pi\frac{doy}{365.25}\right)$$

where *t*_i_ is the observation epoch, *x*(*t*_i_) is the position at *t*_i_ *x*_0_ is the intercept, *v* is the estimated velocity, *doy* is the day of year, and *a*, *b*, *c*, *d* are amplitudes of annual and semi-annual periodic terms^23^.

The uncertainties reported in Table 1 represent 1-*σ* errors estimated via least-squares methods, incorporating temporal correlation effects through appropriate noise modeling. These uncertainties account for: (1) random measurement errors from PPP analysis; (2) temporal correlation in GNSS time series through flicker noise modeling; (3) systematic errors from antenna phase center variations and site-specific effects; and (4) limited observation periods at campaign sites. We incorporated these uncertainties into our error analysis and propagated them through elastic deformation corrections into final GIA component estimates using standard error propagation methods (.

$\:{\sigma\:}_{GIA}=\sqrt{{\sigma\:}_{GNSS}^{2}+{\sigma\:}_{elastic}^{2}}\:$), where $\:\sigma_{GNSS}$ represents the GNSS velocity uncertainty and $\:\sigma_{elastic}$ represents uncertainty in elastic deformation corrections.

### Extraction of GIA signals from GNSS observations using elastic corrections

We analyzed the GNSS observations to isolate the GIA signal by removing the elastic response to present-day changes in ice mass. The observed vertical crustal motion at the GNSS sites consists of two primary components: (1) the instantaneous elastic response to ongoing ice mass changes; and (2) the long-term viscoelastic response to past ice loading (GIA). To separate these components, we first evaluated the elastic deformation using recent mass-change data and subtracted it from the total observed deformation.

The elastic component was calculated using changes in surface mass derived from two independent datasets: (1) GRACE satellite gravity data spanning 2002–2020 (JPL GRACE Mascon Release 06); and (2) satellite altimetry data. For the GRACE-based calculation, we used mascon solutions that had been corrected for GIA effects based on the ICE-6G model^25^. This ensures that our elastic deformation corrections do not introduce double-counting of GIA signals in the final analysis. For the altimetry-based calculation, we converted changes in surface elevation to mass changes using both a firn density model^53^ and the ice density (917 kg/m^3^) to provide upper and lower bounds on the estimates of mass change.

We performed the elastic loading calculation using the Farrell Green’s function method^24^ in the reference frame of the center of mass of the solid Earth (CE). The calculation used a uniform 2.8 × 2.8 km grid mesh across Antarctica, with elastic load Love numbers computed from the Preliminary Reference Earth Model^54^ up to the 10,000th order. The mass change at each grid cell was treated as a disk load, and the total elastic deformation was computed as the sum of the responses to all loads. After calculating both the total observed deformation from the GNSS observations and the elastic response to recent mass changes, we subtracted the elastic component to isolate the GIA signal. This approach allowed us to identify the long-term GIA signal that reflects Earth’s response to past changes in ice mass in the region. The uncertainties in our final GIA estimates incorporate both the GNSS measurement uncertainties and the errors associated with the elastic corrections. We acknowledge potential limitations in our approach, as recent studies^14,55^ have demonstrated that elastic deformation corrections applied to derived rates rather than time series can introduce biases due to mismatches between linear models and correlated noise characteristics.

### Surface-exposure dating at Skarvsnes

The surface-exposure ages used in this study are from Kawamata et al. (2020)^1^, who conducted comprehensive sampling and dating at Skarvsnes. We summarize their methodology here as it provides essential chronological constraints on our GIA modeling.

Rock samples were collected from isolated boulders and cobble-sized erratic rocks positioned stably on bedrock or moraine using a handheld electric cutter with a diamond-tipped blade where necessary. Sample locations and elevations were recorded by GPS, with topographic shielding measured at each site. The samples were processed at the National Institute of Polar Research, Tokyo, Japan, where 40–60 g of clean quartz grains (250–500 μm) were separated for^10^ Be and^26^Al measurements. The isotope ratios were measured at the PRIME Laboratory, Purdue University, West Lafayette, USA, using standard protocols for accelerator mass spectrometry (AMS).

Surface-exposure ages were calculated using the CRONUS-Earth V3 online calculators, employing^10^ Be and^26^Al half-lives of 1.387 Ma and 0.705 Ma, respectively^56–58^. The calculations used CRONUS-Earth production rates with the LSDn scaling scheme^59^, incorporating site-specific scaling factors for latitude, longitude, and elevation. A rock density of 2.7 g/cm^3^ was assumed for all samples, and local topographic shielding corrections were applied. The age calculations include both internal uncertainties from the AMS measurements and external uncertainties related to production rates and scaling. No corrections were made for post-depositional erosion, and GIA-related elevation changes were considered to be negligible (estimated at 1–2%). The resulting^10^ Be ages serve as our primary chronological constraints, with the^26^ Al ages providing supporting data where available.

### GIA modeling

GIA studies in Antarctica have evolved considerably in recent decades. Early research, such as that by Nakada et al. (2000)^60^, focused on modeling relative sea-level (RSL) changes in Antarctica and proposed specific models to explain the observed data (e.g., Okuno and Miura, 2013^61^). Various models of the deglaciation history in Antarctica have since been developed as RSL data and geodetic measurements (e.g., gravity) continue to improve. However, the early models proposed by Whitehouse et al. (2012)^8^, Ivins and James (2005)^26^, and Peltier et al. (2015)^62^ are still widely used to reconstruct the GIA-induced signals. We used the ICE-6G global model of ice-sheet deglaciation^25^ as the starting model in our GIA study and re-examined the mid-Holocene ice-sheet history to better explain the surface-exposure age data for Skarvsnes.

While our study employs a simplified three-layer viscoelastic Earth structure, we recognize that recent advances in GIA modeling have moved toward incorporating 3D Earth structure variations^38,63^. These 3D models can account for lateral heterogeneities in mantle viscosity that may significantly influence predictions of crustal deformation. While our choice of a 1D Earth model is justified for this regional-scale analysis with limited spatial extent, we acknowledge that larger-scale or continent-wide studies would benefit from incorporating a 3D Earth structure. The good agreement between our model predictions and observations suggests that, for this particular region, a well-calibrated 1D Earth model can adequately capture the first-order GIA response; however, future studies incorporating a 3D Earth structure may provide additional insights into the finer details of mantle heterogeneity and its influence on regional crustal deformation patterns.

The viscoelastic structure beneath the Antarctic continent also has a key role in GIA modeling. Seismological data suggest the presence of continental-scale horizontal heterogeneities in the viscosity structure beneath Antarctica. Several studies have investigated these heterogeneities using various seismic datasets, such as surface-wave dispersion^32^ and shear-wave splitting^64^. The results indicate lateral variations in the upper-mantle viscosity structure beneath Antarctica, with implications for GIA-induced signals^38^. In addition, GIA modeling to explain the GRACE data, including the lateral variation in viscosity structure beneath Antarctica, is important for the precise interpretation of the ice mass balance of Antarctica^27,65^.

Although Antarctic GIA-induced signals are affected by lateral variations in the internal structure of the Earth, GNSS observations were used in this study, which focus on the local observational area in the Lützow–Holmbukta region. GNSS observations are considered to be minimally affected by lateral variations in Earth’s structure at a given observation site. Therefore, we adopted a three-layer viscoelastic structure for our GIA predictions, consisting of elastic lithosphere, upper mantle, and lower mantle (Fig. S2). The thickness of the elastic lithosphere and the upper- and lower-mantle viscosities were varied over ranges consistent with globally proposed models. We performed numerical calculations using a suite of Earth models with different lithospheric thickness and mantle viscosity combinations to assess the sensitivity of our GIA predictions to the underlying viscoelastic structure.

We varied the lithospheric thickness from 50 to 120 km based on seismological constraints^31–33^. We considered upper-mantle viscosity values between 5 × 10^20^ and 1 × 10^21^ Pa s to encompass the estimates from previous Antarctic GIA studies^8,26^ and global viscosity models^34–36^. We varied the lower-mantle viscosity from 10^21^ to 10^23^ Pa s based on the range of estimates from global studies^34–36^. These radial viscosity profiles are shown in the Supplementary Information (Fig. S2).

To comprehensively explore the parameter space governing Earth’s viscoelastic response to ice-loading changes, we systematically tested 432 distinct viscosity structure combinations. These combinations were derived by varying the lithospheric thickness (50–120 km), upper-mantle viscosity (5 × 10^20^ to 1 × 10^21^ Pa s), and lower-mantle viscosity (10^21^ to 10^23^ Pa s) across physically plausible ranges. Each of these Earth model configurations was then evaluated against 20 ice-loading histories, including the original ICE-6G model and our custom scenarios (RM1, RM2, RA1, RA2, and others not presented in the main text) that incorporate varying magnitudes of mid-Holocene rapid thinning and potential re-thickening. This systematic parameter exploration, which yielded thousands of unique model scenarios, enabled us to robustly assess the sensitivity of predicted crustal deformation patterns to both Earth rheology and ice loading history, while acknowledging the inherent limitations of our 1D Earth model framework.

We explored this parameter space and compared the modeled GIA response, incorporating the rotational feedback^66^ with the observed rates of surface deformation and surface-exposure age data to constrain the viscoelastic structure beneath the Lützow–Holmbukta region and improve our understanding of the regional GIA signal. This approach allowed us to assess the effect of the Earth model on the inferred ice-loading history. It also provided a framework for investigating the sensitivity of our results to the underlying viscoelastic structure.

### Statistical analysis and model comparison

To quantitatively evaluate model performance and establish statistical significance of improvements, we employed several statistical measures and hypothesis tests. The weighted root-mean-square ($\:WRMS$) residual between observed and predicted GIA rates was calculated as:$\:WRMS=\sqrt{\frac{\sum_{i=1}^{n}{\left(\frac{\text{y}_{i,obs}-\text{y}_{i,pred}}{\sigma_{i}}\right)}^{2}}{n}}$

where $\:{\text{y}}_{i,obs}$ and $\:{\text{y}}_{i,pred}$ are the observed and predicted uplift rates at site *i*, $\:\sigma_{i}$ is the observational uncertainty at site *i*, and *n* = 3 is number of observation sites.

The goodness-of-fit was evaluated using the *c*-squared statistic:

$$\:\chi^{2}=\sum\limits_{i=1}^{n}{\left(\frac{\text{y}_{i,obs}-\text{y}_{i,pred}}{\sigma_{i}}\right)}^{2}$$

*F*-tests were performed to assess whether model improvements over the ICE-6G baseline were statistically significant. The *F*-statistic was calculated as:

$$\:F=\frac{\left(\chi_{\text{ICE-6G}}^{2}-\chi_{\text{improved}}^{2}\right)/1}{\chi_{\text{improved}}^{2}/2}$$

Where the degrees of freedom are $\:{\nu}_{1}=1$ (numerator) and $\:{\nu}_{2}=2$ (denominator, calculated as $\:\text{n}\:-\:1$ where $\:\text{n}\:=\:3$ is number of observation sites).

The corresponding *p*-value was calculated using the cumulative *F*-distribution:

$$\:p=1-F_{CDF}\left(F;\text{1,2}\right)$$

Where $\:{F}_{CDF}$ is the cumulative F-distribution function with degrees of freedom (1, 2). Statistical significance was determined at the ${\alpha}=\:0.05$ level, where $\:p\:<\:{\alpha\:}$ indicates statistically significant model improvement.

## Supplementary Information

Below is the link to the electronic supplementary material.

Supplementary Material 1

## Data Availability

All calculation results presented in this study are available from the Arctic Data Archive System (ADS) at 10.17592/001.2025101701^67^.The simulation codes for the GIA modeling can be obtained from the corresponding author upon reasonable request.
