# Supplementary material for: Mid Holocene rapid thinning and rethickening of the East Antarctic ice sheet suggested by glacial isostatic adjustment
Source: Sci Rep. 2025 Nov 17;15:40207. doi: 10.1038/s41598-025-24176-4 (PMC12623747; doi:10.1038/s41598-025-24176-4)
Supplement: Supplementary file 1 — Supplementary Material 1 [file 41598_2025_24176_MOESM1_ESM.pdf]

Supplementary Information for "Mid Holocene Rapid Thinning and Rethickening of the East Antarctic Ice Sheet Suggested by Glacial Isostatic Adjustment"

Okuno et al.

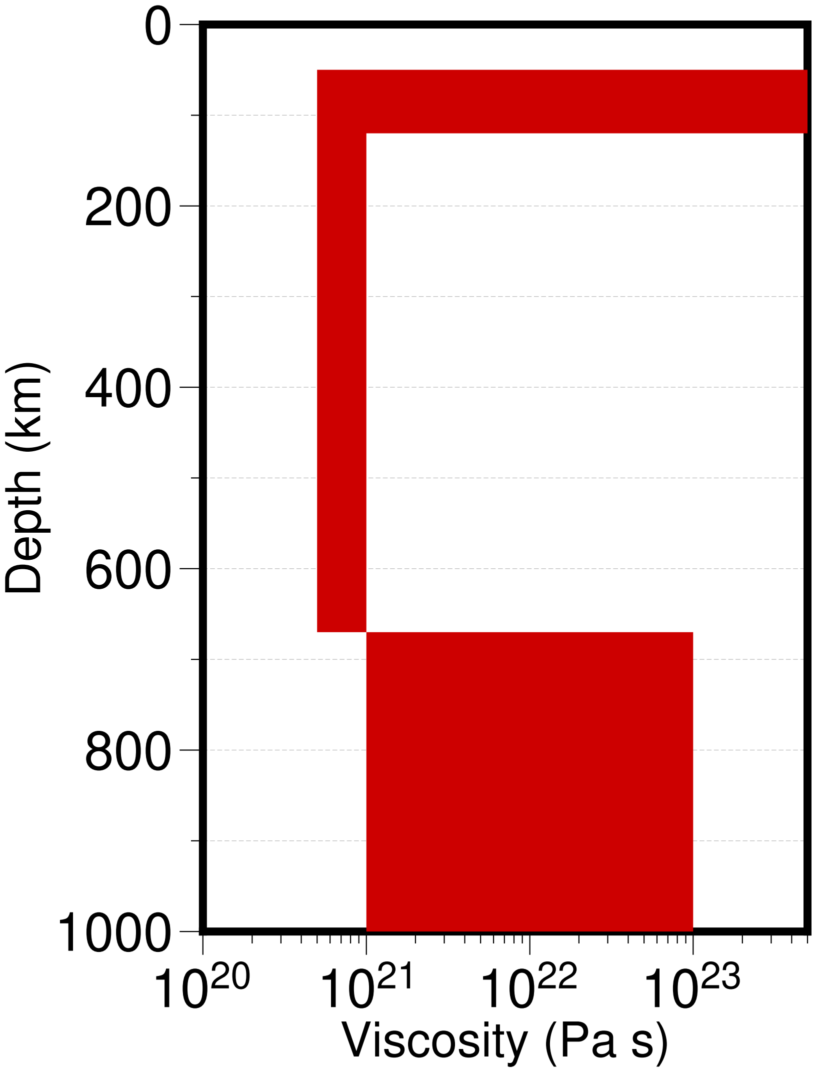

Figure S1. Radial viscosity profiles adopted in this study. The elastic lithospheric thickness was varied from 50 to 120 km. The upper-mantle viscosity ranges between 5×10^{20} and 1×10^{21} Pa s, while the lower-mantle viscosity varies from 10^{21} to 10^{23} Pa s.

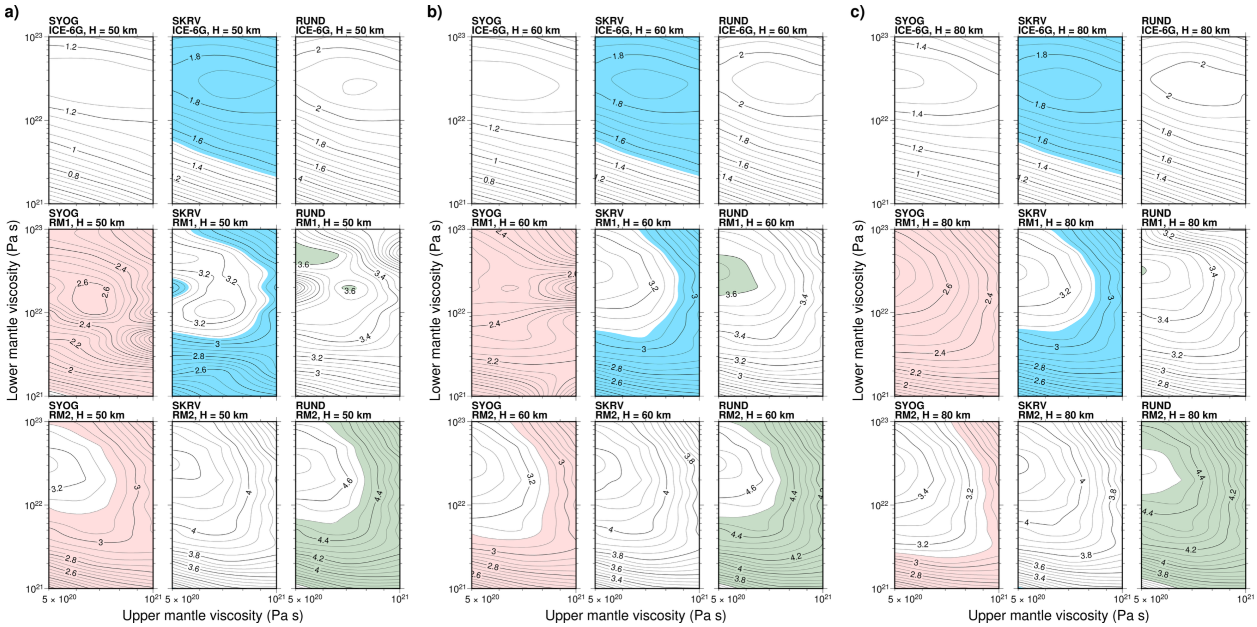

Figure S2. Predicted rates of crustal deformation at each GNSS site as a function of the upper- and lower-mantle viscosities. The lithospheric thicknesses, whereby the top layer behaves as the elastic layer, are **(a)** 50 km, **(b)** 60 km, and **(c)** 80 km for all the viscosity models depicted here, and the results for a 70 km elastic thickness are shown in Fig. 6. The color-shaded regions indicate the permissible ranges constrained by the GNSS observations at each site, and there are no composite areas that satisfied all observation sites for each ice model. ICE-6G, RM1, and RM2 were used as the models of ice sheet history.

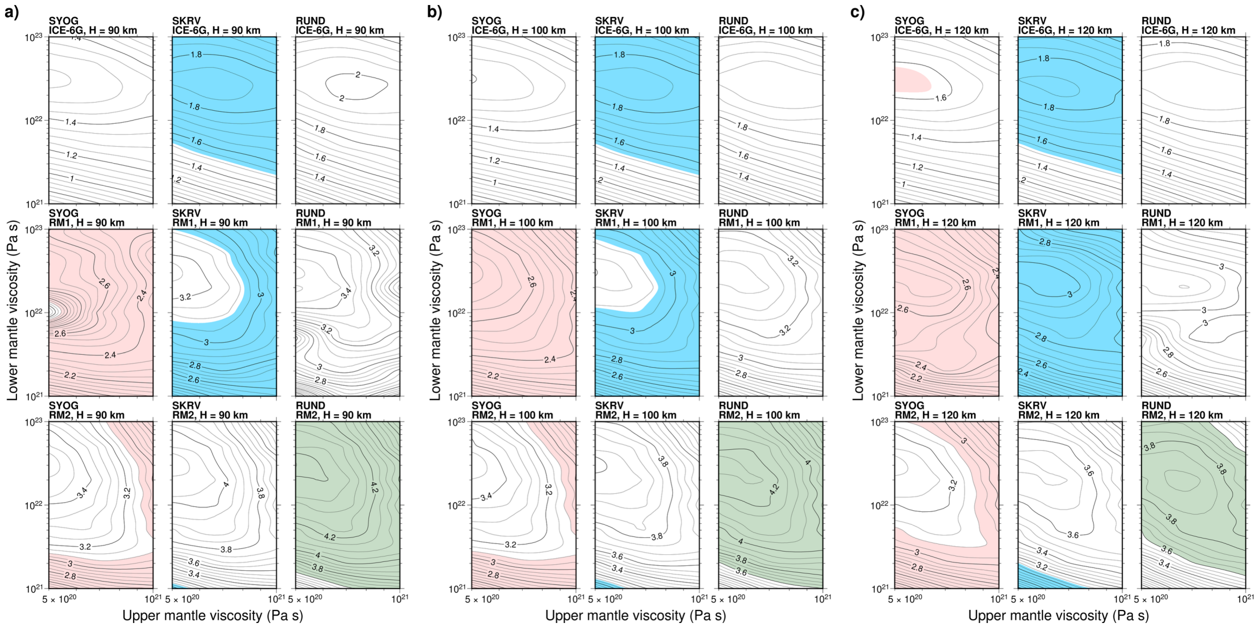

Figure S3. As for Fig. S2, but with elastic thickness models of **(a)** 90 km, **(b)** 100 km, and **(c)** 120 km. These results also show that there are no composite areas that satisfy all observation sites for each ice model.

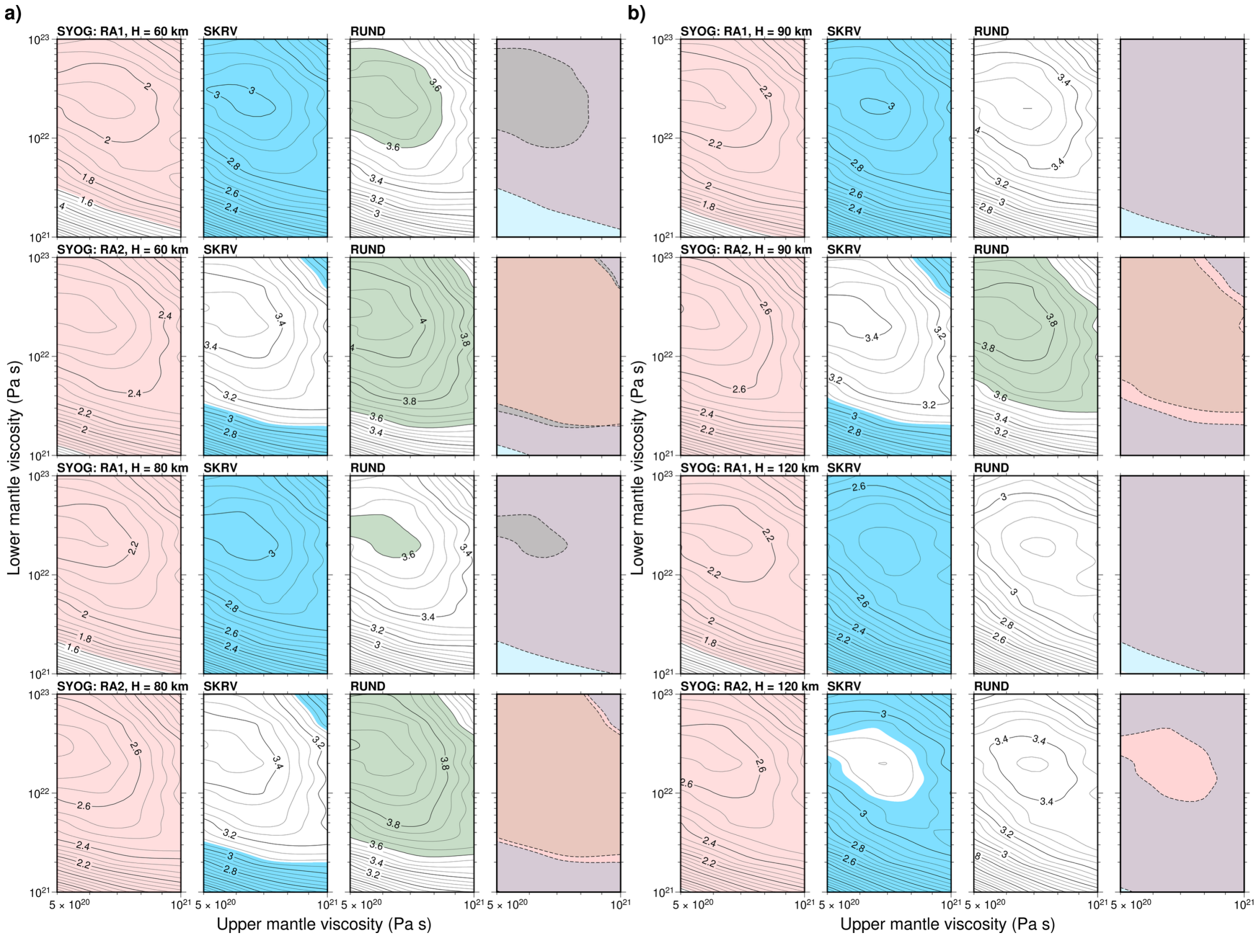

Figure S4. Predicted rates of crustal deformation at each site as a function of upper- and lower-mantle viscosities. Adopted lithospheric thicknesses of **(a)** 60 and 80 km, and **(b)** 90 and 120 km are depicted here, and the results of other models of elastic thickness are shown in Fig. 9. The color-shaded regions indicate the permissible ranges constrained by the GNSS observations at each site. RA1 and RM2 were used here as the models of ice sheet history.

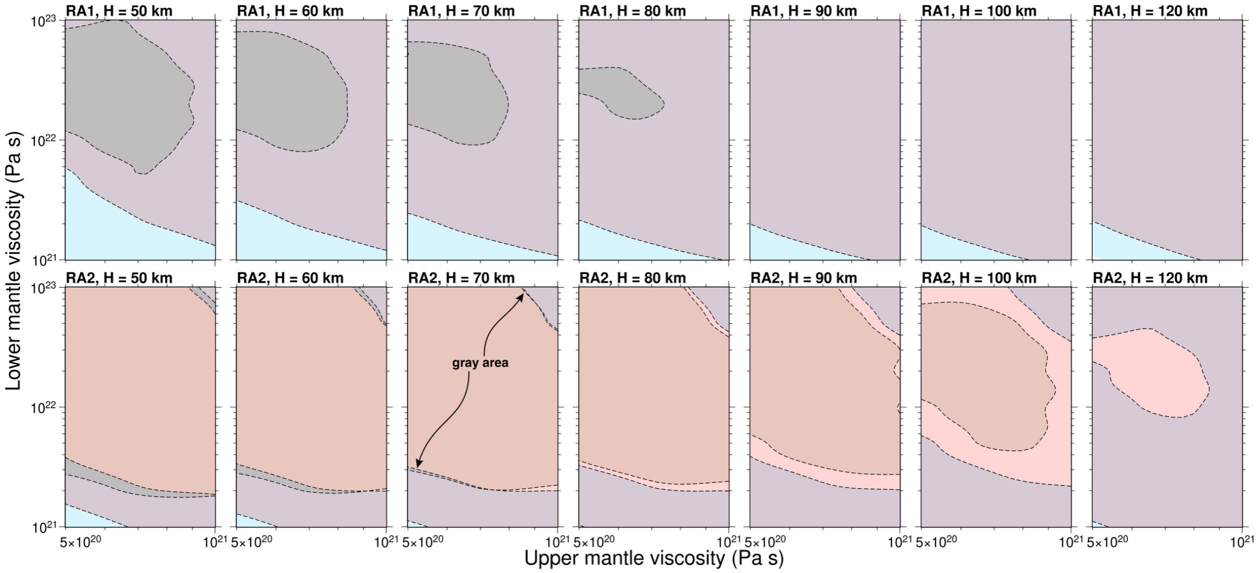

Figure S5. Permissible mantle viscosity ranges, as illustrated in Figs. 9 and S4, which satisfy the constraints imposed by GNSS observational data and GIA predictions at all sites. Gray areas indicate solutions incorporating ice history models with re-thickening scenarios (RA1, RA2). Models with lithospheric thickness exceeding 90 km fail to produce solutions consistent with observational constraints across all sites.
